# Shape Memory Polymer Foam Based on Nanofibrillar Composites of Polylactide/Polyamide

**DOI:** 10.3390/molecules29215045

**Published:** 2024-10-25

**Authors:** Dhanumalayan Elumalai, Ramin Hosseinnezhad, Vladislav Bondarenko, Jerzy Morawiec, Iurii Vozniak, Andrzej Galeski

**Affiliations:** 1 Centre of Molecular and Macromolecular Studies, Polish Academy of Sciences, Sienkiewicza Str., 112, 90363 Lodz, Poland; dhanumalayan.elumalai@cbmm.lodz.pl (D.E.); ramin.hosseinnezhad@cbmm.lodz.pl (R.H.); jerzy.morawiec@cbmm.lodz.pl (J.M.); andrzej.galeski@cbmm.lodz.pl (A.G.); 2 Physics and Mathematics Department, Kryvyi Rih State Pedagogical University, Gagarin Av. 54, 50086 Kryvyi Rih, Ukraine; vladislavb167@gmail.com

**Keywords:** shape memory, polymer foams, polymer–polymer nanocomposites, supercritical carbon dioxide foaming

## Abstract

This paper presents the novel development of a shape memory polymer foam based on polymer–polymer nanocomposites. Herein, polylactide (PLA)/biosourced polyamide (PA) foams are fabricated by in situ fibrillation of polymer blends and a subsequent supercritical CO_2_ foaming technique. In this system, PLA serves as a shape memory polymer to endow this foam with a shape memory effect (SME), and in situ generated PA nanofibers are employed to reinforce the PLA cell walls and provide an additional permanent phase. A concentration of PA, 5 wt.%, was chosen to form an entangled nanofibrillar network. Foams of PLA/PA nanoblends with the same content of constituents were fabricated to reveal the effect of minor phase morphology on the cell structure and shape memory behavior of polymer foams. Profiting from the reinforcing effect of PA nanofibers, the PLA/PA nanocomposite foam exhibits smaller foam cells, a narrower cell size distribution and a comparable cell concentration than the PLA/PA nanoblend foam. In addition, PA nanofibers, unlike PA nanodroplets, favor the shape fixation ratio and recovery ratio and shorten the shape recovery time.

## 1. Introduction

Shape memory polymer foams are a new class of intelligent polymer materials that have a porous structure and combine the characteristic features of conventional foams and shape memory polymers (SMPs) [1,2,3,4,5]. The advantages of shape memory polymer foams are their lower specific weight and faster recovery compared with SMPs. Furthermore, by adding functional additives, additional functional properties of foams can be achieved, such as fire resistance, heat resistance, self-healing, etc. [6,7,8,9,10]. 

Shape memory polymer foams are produced either on the basis of thermoplastic foams [11,12,13,14,15] or on the basis of thermoset foams [16,17,18,19]. The commonly used thermoplastic foams are easy to process and recyclable, but have poorer mechanical properties, while thermoset foams have better thermomechanical properties, but are difficult to process due to the cross-linking of their macromolecular structures. There are also polymer foams, which are a combination of thermoplastics and thermosets. However, their properties depend in a complex way on the multi-level hierarchical structure that forms and are currently poorly understood [20]. Nevertheless, the possibility of combining the advantages of thermoplastics and thermosets in a foamed polymer material is very promising.

The properties of polymer foams depend not only on the polymer material from which they are made but also on the foaming process. The most environmentally friendly and therefore widely used process is foaming with supercritical carbon dioxide (sc-CO_2_). However, the use of sc-CO_2_ as physical foaming agent has a number of limitations. In particular, it is ineffective for thermoplastics with high melt viscosity but low thermal stability (polyvinyl chloride) or high processing temperatures close to the decomposition temperature (polycarbonate, poly(phenylene oxide)) [11,21]. The addition of other polymers or, more effectively, a low molecular weight plasticizer, is often used to reduce the melt strength (or viscosity) so the foaming process can be carried out at a lower temperature. However, the presence of plasticizers often carries the risk of reducing the mechanical strength and heat resistance of the resulting foams. In the case of thermosetting plastics, the curing and foaming processes must take place simultaneously. In this case, cell growth requires a suitable viscosity, but the viscosity of thermosets, which depends not only on the foaming temperature but also on the degree of pre-curing, rises close to the gel point in a very short time during the curing process [22,23,24,25]. Recently, significant progress has been made, particularly, in producing rigid thermosetting microcellular polyurethane foams with medium-to-low densities using physical blowing agents such as N_2_ and CO_2_ gas [24,26,27,28,29].

The formation of shape memory foams requires two structural components, including a reversible switching phase and a permanent phase [30]. This usually requires the creation of more complex systems consisting of phases responsible for the transition of the shape memory and phases responsible for the storage of the permanent/temporary shapes. However, in both thermoplastics and thermosets with more complex, e.g., two-phase or multi-phase structures, the interphase region can be a pathway for CO_2_ escape or an obstacle to efficient foam expansion, as well as lead to the formation of undesirable open porosity. In such systems, attempts are made to separate the foaming and phase separation processes. Several strategies have been developed for this purpose. In the first strategy, called “foaming-reinforcing”, a cellular structure is formed and then microphase separation is carried out in the resulting cell walls [20]. The second strategy is a combination of preformed large-pored flexible foam and solid–liquid phase change materials via impregnation [31,32]. However, the search for alternative ways to produce shape memory foams with low weight and high performance is urgently needed.

Generally, the introduction of a light and strong filler, such as carbon nanotubes or carbon nanosheets, is used to increase the strength of polymer foams as well as to improve their foamability [33,34,35,36,37,38,39,40]. An effective improvement in their mechanical response and rheological behavior during extensional deformation is achieved by the formation of an effective interphase. However, the interphase layer formed between the polymer matrix and the filler, which is a network of physical entanglements, can also serve as a permanent phase to enable polymers with shape memory effect [41,42]. From this point of view, polymeric fillers seem to be the most promising as they exhibit stronger adhesion to the polymer matrix and do not require additional functionalization [43]. Compared with polymer nanodroplets, polymer nanofibers form a higher phase continuity, which contributes to higher shape memory properties (shape fixation and shape recovery ratio) [44]. Furthermore, the use of in situ generated polymer nanofibers formed during the conversion of polymer nanoblends into polymer–polymer nanocomposites, also known as nanofibrillar composites [45,46,47,48], would allow the greatest impact in terms of forming an effective interphase layer, as they are characterized by a homogeneous distribution and high aspect ratio compared with ready-made polymer nanofibers [49,50,51]. Polymer nanofibers can also form a physical network between themselves that can induce or enhance strain hardening behavior, also known as extensional thickening, during cell growth, facilitating the production of a closed-cell structure with a high expansion ratio and uniform cell distribution [52]. To our knowledge, few papers [52,53] have investigated the possibility of using in situ generated polymer–polymer nanocomposites to form polymer foams. At the same time, the shape memory performance of such foams has not yet been investigated.

In this study, the possibility of forming polymer foam with shape memory via the sc-CO_2_ foaming process of polymer–polymer nanocomposites is demonstrated using the polylactide (PLA)/biosourced polyamide (PA) system as an example. The effects of the morphology of the minor polymer phase (droplet or fiber network) on the foam formation and shape memory performance were investigated.

## 2. Results and Discussion

### 2.1. Cell Structure of PLA/PA Foams

**Figure 1:**
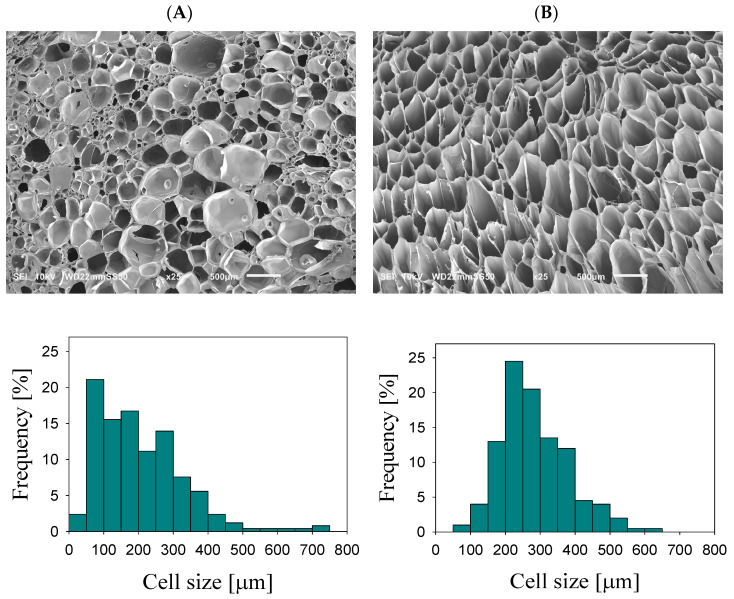
SEM micrographs of the foams of PLA/PA nanoblends (**A**) and in situ generated nanocomposites (**B**) with corresponding diagrams of cell size distribution.

Figure 1A,B shows SEM images of the foamed PLA/PA nanoblends and in situ generated nanocomposites with the corresponding cell size distribution diagrams. The foamed PLA/PA nanocomposite showed a homogeneous cell structure with an average cell size of 280 μm and a unimodal distribution. At the same time, the foamed PLA/PA nanoblend showed a more complex cell structure with a bimodal cell size distribution and the presence of large cells with an average cell size of 316 μm and small cells with an average cell size of 70 μm. The cell concentration is slightly higher in the foamed nanoblend than in the foamed nanocomposite: 1.78 × 10^6^ cells/cm^3^ and 0.88 × 10^6^ cells/cm^3^, respectively. However, the contribution of a large number of small cells located at the edge of the intersection of large cells to the obtained cell concentration value should be taken into account.

**Figure 2:**
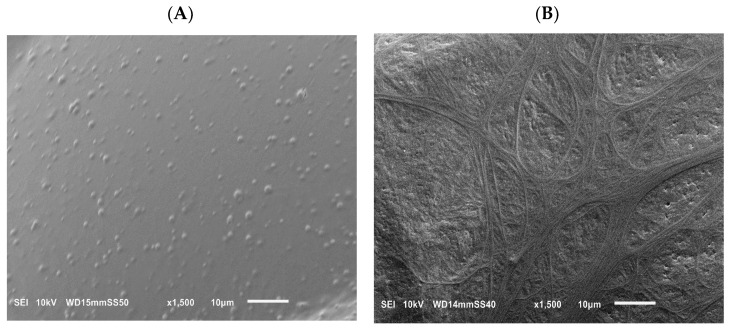
SEM micrographs of the surfaces of foamed PLA/PA nanoblends and in situ generated nanocomposites showing the PA nanodroplets (**A**) and PA nanofibers (**B**) in the reinforced cell walls of PLA matrix.

In Figure 2A, the PLA cell walls contain inclusions of nanodroplets that are homogeneously distributed in the polymer matrix but do not form additional physical bonds, whereas the foams of composite clearly show the presence of a large number of PA nanofibers in the PLA cell walls (also comparatively seen in Figures S1c,d and S2c,d provided in the Supplementary Materials). In addition, the PA nanofibers form a spatial network with many physical entanglements (Figure 2B). As mentioned in [52], the entangled network of polymer nanofibers appears to play a crucial role in reinforcing the growing cells. First, the polymer nanofibers localized in the cell walls induce strain hardening (Figure S3 provided in the Supplementary Materials), which in turn prevents the cells from breaking during expansion. As a result, the phenomenon of coalescence was suppressed in the PLA/PA nanocomposite foams. The coalescence of growing cells during sc-CO_2_ foaming of the PLA/PA blend leads to the generation of foams with a much lower concentration of large cells. Secondly, the entanglements between the polymer nanofibers appear to act as a local confinement for the further expansion of the growing cells. Moreover, the network of PA nanofibers favors efficient stress distribution during cell formation, which contributes to the formation of cells with more uniform sizes.

### 2.2. Mechanical Properties of PLA/PA Foams

**Figure 3:**
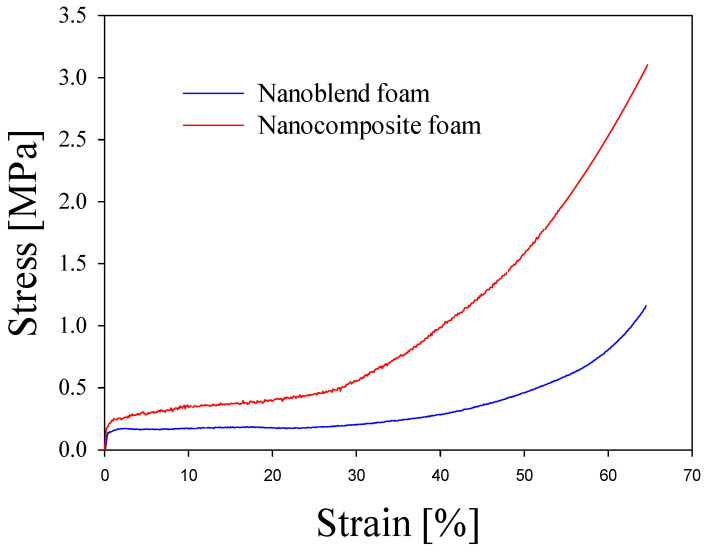
Representative stress–strain curves determined for sc-CO_2_ foam of PLA/PA nanoblends and in situ generated nanocomposites deformed at room temperature: in the plane-strain compression.

The mechanical properties of nanoblend and nanocomposite foams are closely related to their cellular microstructures, including cell density and size, wall thickness and surface defects, as well as the morphology of nanoparticles. Figure 3 shows the compression behavior of the foams. The corresponding mechanical characteristics are detailed in Table 1. Both foams respond initially with elastic response reaching the level of 0.2 MPa. The modulae for both foams are different: 0.278 MPa for the nanoblend foam and 0.466 MPa for the nanocomposite foam. With farther compression, the nanocomposite foam responds with significantly larger stress 0.30–0.35 MPa while the nanoblend foam is much softer, at the level of 0.20–0.25 MPa. With further compression, both foams respond with evident strain hardening, with nanocomposite foam responding with approximately twice the stress for nanoblend foam. This is probably due to the stronger reinforcing effect of the PLA matrix caused by the presence of PA nanofibers compared with PA nanodroplets [54,55,56,57,58,59,60,61]. The influence of the cell structure can also be observed. Cell walls, ribs at junctions of cells and cell corners have a major influence on the mechanical properties of a foam. They all are stronger and fortified by PA nanofibers in the case of nanocomposite foam than for nanoblend foam, resulting in a higher modulus and higher strength. In addition, a more uniform distribution of cell sizes should contribute to better overall mechanical properties (compare Figure 1A,B). It should be noted that some defective wall surfaces were formed in the nanoblend foams and the wall thickness was slightly less than the wall thickness in the nanocomposite foams, which also contributed to the strength of the foams.

### 2.3. Shape Memory Properties of PLA/PA Foams

In the PLA/PA polymer system, the amorphous phases of PLA can be selected as the switching elements, while the crystalline phases of the PA and PLA/PA interphase serve as the permanent elements. The amorphous phase of PA can potentially be a switching element, but its low volume content does not allow efficient accumulation of elastic energy to realize the shape memory effect. The glass transition temperature of PLA, 60 °C, was chosen as the programming temperature for the shape memory effect.

**Figure 4:**
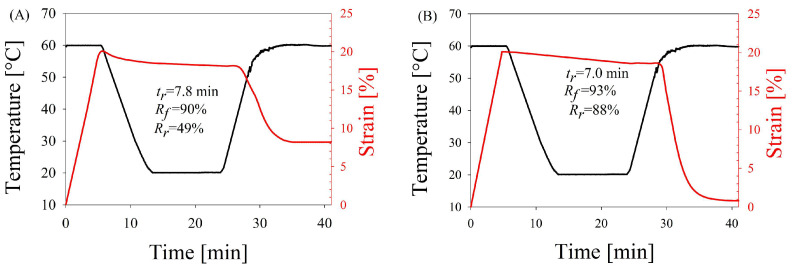
Temperature and strain of foams of PLA/PA nanoblends (**A**) and in situ generated PLA/PA nanocomposites (**B**) during shape memory cycle at deformation temperature (T_d_) of 60 °C.

Figure 4 shows typical stress–strain-temperature curves as a function of time for nanoblend and nanocomposite foams. The transition temperature was set at 60 °C, with an overall strain of 20%. As can be seen, both nanoblend and nanocomposite foams exhibit relatively high shape fixation ratios, namely 90 and 93%, respectively. At the same time, the shape recovery ratios differ significantly from each other, being 88% for nanocomposite foam and only 49% for nanoblend foam. The shape recovery times were similar but slightly shorter for the nanocomposite foam (7.0 min for nanocomposite foam vs. 7.8 min for nanoblend foam). Table 1 summarizes the shape memory characteristics, while Figure S4 provided in the Supplementary Materials displays real images illustrating the permanent, temporary and recovered shapes of PLA/PA nanoblend and in situ generated composites.

**Figure 5:**
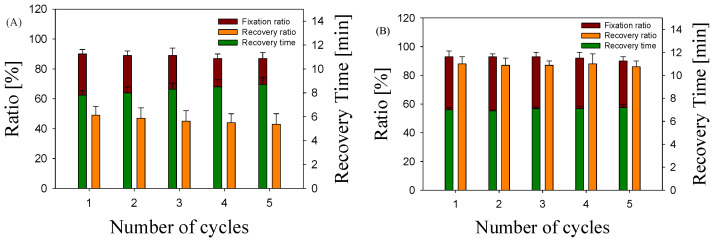
Shape fixation and shape recovery ratios as well as shape recovery time of PLA/PA nanoblend foams (**A**) and PLA/PA nanocomposite foams (**B**) for different numbers of cycles.

In [62,63], it was found that the expansion ratio of the foam (directly proportional to the cell concentration) and the cell size both affect the memory performance. In particular, when the expansion ratio of the foam is increased, the shape fixation ratio increases and the shape recovery ratio decreases. At the same time, when the cell size of the foam is reduced, the shape fixation ratio decreases and the shape recovery ratio increases. In the systems studied, reducing the large cell size actually led to an increase in the recovery ratio, and nanocomposite foams with a lower cell concentration showed a higher recovery ratio. The shape fixation ratio was practically independent of the cell microstructure parameters. It can be assumed that the cell structure had no decisive influence on the properties of the shape memory effect of the foams studied. In this case, the morphology of the polymer–polymer system from which the foam was produced had a greater influence. The presence of entangled PA nanofibers acting as permanent elements contributed to a more efficient accumulation of elastic deformation and to a significantly lower irreversible deformation in switching PLA elements compared with PA nanodroplets as permanent elements. It is also noteworthy that the deterioration of shape memory properties during cycling (shape fixation and shape recovery ratios, recovery time) was practically absent in the nanocomposite foams, while it was observed in the nanoblend foams (Figure 5). The latter could also be related to the presence of an entangled nanofibril network in the foam walls, which effectively redistributes the load during shape memory effect programming and shape recovery [44]. 

## 3. Materials and Methods

### 3.1. Materials

A commercially available grade of polylactide, PLA 4060D, was purchased from NatureWorks LLC and used as the matrix. This grade of PLA is biodegradable, has a density of 1.24 g cm^−3^ and a molecular weight of 120,000 g mol^−1^. It has a glass transition temperature of 61 °C. A fully bio-based polyamide (PA) with the trade name Vestamid Terra DS from Evonik Industries was used to produce the composite materials. The monomers for the synthesis of this type are obtained from castor oil. The melting point of PA is 200 °C and its equilibrium melting temperature is 238 °C.

### 3.2. Sample Preparation

Blends and in situ generated nanocomposites of PLA/PA were prepared according to the procedure we have already described [64]. To produce the blend PLA/PA (95/5), 5 wt.% PA was melt-blended with PLA (both components were dried for 8 h at 60 °C) in a twin-screw extruder, whereby the temperature zones were progressively adjusted from 200 °C to 230 °C. The blend was also produced in a single-screw extruder. The mixture was also processed in a single-screw extruder with a temperature gradient from 230 °C (feed zone) to 175 °C (slit nozzle). In situ generated fibrillar nanocomposites of PLA/PA were processed using a co-rotating twin-screw extruder 2 × 20/40D EHP (Zamak Mercator) operating at 120 rpm. Controlled shear extrusion processes were achieved through the optimization of screw rotation speed (ω), and temperature. After fibril formation, the tapes were extruded using a single-screw extruder (PlastiCorder PLV 151, Brabender; D = 19.5 mm; L/D = 25 and 20 rpm), which was equipped with a 12 mm wide, 0.8 mm thick and 100 mm long slit die. The slit die process pressure of the slit nozzle was 20.1 MPa. Upon shearing, the zone temperature of the extruder barrel was set accordingly to induce the crystallization process of the deformed minor phase (i.e., PA). The extrudates exited from the single-screw extruder were cast on a transport belt at ambient temperature without additional drawing. The extrudates were obtained as tapes ~0.5 mm thick and 10 mm wide. Figure S5 provided in the Supplementary Materials presents a schematic depicting the transformation of droplets into fibers and their stabilization under aa high shear rate during extrusion. The PLA/PA mixture shows a typical two-phase structure with nanoscale PA particles (Figure S6a provided in the Supplementary Materials). The average particle size is 847 nm. In contrast, the PLA/PA composite produced in situ exhibits a fibril morphology of the dispersed phase (Figure S6b provided in the Supplementary Materials). PA nanofibrils with an average diameter of 490 nm are formed, which are randomly oriented, increasing the probability of physical entanglements between the nanofibers and the formation of a nanofibril network.

### 3.3. Foaming Process

Before initiating the foaming process, the as-prepared blend and in situ generated composites were hot-pressed in a hydraulic press using a steel template in the form of rectangular plates with dimensions of 8 × 5 cm in length and width and a thickness of 1 mm. Further, round disks with a diameter of 20 mm were cut from the plates and used for foaming. The foams of PLA/PA blends and composites were produced in a high-pressure stainless steel autoclave reactor using a batch foaming process. The reactor vessel was preheated to 100 °C. The samples were filled into an aluminum pan and loaded into the reactor vessel (50 mL). The reactor vessel was then filled with dry ice and sealed. The foaming temperature was set to 150 °C for the PLA/PA blend and 155 °C for the PLA/PA composite samples. The CO_2_ dissolution pressure was maintained at 13 MPa and the soaking time was 60 min for all the samples. The autoclave was gradually cooled to 120 °C for the blends and 130 °C for the composites, and then the pressure was quickly released (~1 s) via the vents in the autoclave chamber. The foamed samples were immediately quenched with dry ice and stored for analysis. This experimental procedure was repeated several times for each composition of the samples.

### 3.4. Apparatus and Experimental Techniques

The structure of the foams was analyzed by scanning electron microscopy (SEM) using a JEOL JSM 6010 LV/LA microscope. The internal structure of the samples was visualized by cutting at liquid nitrogen temperature. The cell size and cell concentration of foams were determined from SEM images. The number of cells (N_0_) per unit volume (cm^3^) of the foamed samples was calculated using the following formula

(1) $$N_{0}={\left(\frac{n}{A}\right)}^{\frac{3}{2}}\left(\varphi \right)$$

where *n* is the number of cells and *A* is the area (cm^2^) of the SEM micrograph. The foaming expansion ratio (φ) is the ratio of the density of the solid sample (ρ_0_) to the density of the foamed sample (*ρ_f_*) and is given as follows,

(2) $$\varphi =\frac{\rho_{0}}{\rho_{f}}$$

Uniaxial extension tests on samples were conducted using an extensional viscosity fixture (EVF) attached to the ARES rheometer. Rectangular specimens, measuring 18 × 10 × 0.7 mm³, were either prepared by hot compression molding at 200 °C in a standard mold (for blend) or cut from extruded tapes (for composite). These specimens were stretched uniaxially at 160 °C with constant Hencky strain rates of 0.05 and 0.5 s⁻^1^. The temperature was adjusted to ensure that the elastic force of the minor phase was strong enough to counteract the plastic flow of the molten matrix. At higher temperatures, the effect of nanofibers would be masked due to the dominant plasticity of the major phase. The tensile stress growth coefficient, η^+^_E_ (t, ε_H_), as a function of strain time (t) at a given Hencky strain rate (ε_H_), was recorded.

Thermally activated shape memory characterization of foamed samples was conducted using a Q800 DMA instrument with the film tension clamp under controlled strain and controlled force modes. The dual shape memory properties were enforced by a primary strain of 20% applied at 60 °C. The strain was then held constant while the sample was cooled to room temperature and then released. To observe the memory effect, the samples were heated to 62 °C for 30 min to recover the shape.

The shape fixity R_f_ and the shape recovery R_r_ ratios were calculated using the following equations: *R*_*f*_ = (ε_*un*_/ε_*max*_) × 100%; *R*_*r*_ = ((ε_*un*_−ε_*fin*_)/ε_*max*_) × 100%, where ε_un_ is the strain after cooling and unloading, ε_max_ is the strain obtained before the constant loading was released and ε_fin_ is the strain obtained after heating in the step of recovery.

The mechanical properties of the foamed samples were tested under plane-strain compression using the Instron 5582 tensile testing machine. The test temperature was 25 °C. The specimen was compressed at a rate of 5% of the initial thickness per minute. The specimens had a rectangular shape with a size of 8 × 4 × 3 mm.

## 4. Conclusions

The concept of in situ generated polymer–polymer nanocomposites was applied to the production of shape memory polymer foams. In situ generated polymer nanofibers are characterized by a high degree of dispersion in the polymer matrix and an associated high specific surface area, which significantly improves the adhesion between the phases in polymer–polymer systems. Furthermore, in situ generated polymer fibers are able to form entangled nanofiber structures due to the end-to-end coalescence process. In situ generated nanofibers not only have a positive effect on the foaming behavior by ensuring the formation of a homogeneous, closed cell structure with uniform cell size distribution, but also on the improvement of mechanical properties and shape memory. The effects achieved are due to the formation of an effective interphase adhesion between major and minor polymer phases. Using the PLA/PA system as an example, it is shown that the foam produced on the basis of an in situ generated polymer–polymer nanocomposite has excellent shape memory properties (shape fixation of 93% and recovery of 88%), which are significantly higher than that of the foam based on a polymer–polymer nanoblend (shape fixation of 90% and recovery of 49%). In addition, nanocomposite foams have higher mechanical properties (modulus of elasticity of 0.466 MPa, yield stress of 0.30 MPa) compared with nanoblend foams (modulus of elasticity of 0.278 MPa, yield stress of 0.20 MPa).

## Figures and Tables

**Table 1:** Mechanical and shape memory properties of nanoblends and in situ generated composites of PLA/PA.

PLA/PA	Modulus of Elasticity, MPa	Yield Stress, MPa	Shape Fixation Rate,%	Shape Recovery Rate,%	Shape Recovery Time, min
Nanoblend	0.278	0.20	90	49	7.8
Nanocomposite	0.466	0.30	93	88	7.0

## Data Availability

The original contributions presented in this study are included in the article, and further inquiries can be directed to the corresponding author.

## Supplementary Materials

The following supporting information can be downloaded at: https://www.mdpi.com/article/10.3390/molecules29215045/s1, Figure S1 (a–d): SEM micrographs of cross-section surfaces of foams of PLA/PA nanoblends. Figure S2 (a–d): SEM micrographs of cross-section surfaces of foams of PLA/PA in situ generated nanocomposites. Figure S3: Time-dependence of the tensile stress growth coefficient, η+E (t, εH), for PLA/PA nanoblend and in situ generated nanocomposite. Figure S4: Illustration of the shape memory behavior of PLA/PA nanoblend and in situ generated composites. Figure S5: Schematic illustrating the transformation of droplets into fibers and their stabilization under high shear rate during extrusion. Figure S6: SEM images of PLA/PA nanoblend (left) and in situ generated composites (right) at higher magnification of 4500X and histograms of PA particle size distribution.
