# Supplementary material for: Shape Memory Polymer Foam Based on Nanofibrillar Composites of Polylactide/Polyamide
Source: Molecules. 2024 Oct 25;29(21):5045. doi: 10.3390/molecules29215045 (PMC11547612; doi:10.3390/molecules29215045)
Supplement: Supplementary file 1 [file molecules-29-05045-s001.zip › molecules-3244722-supplementary.pdf]

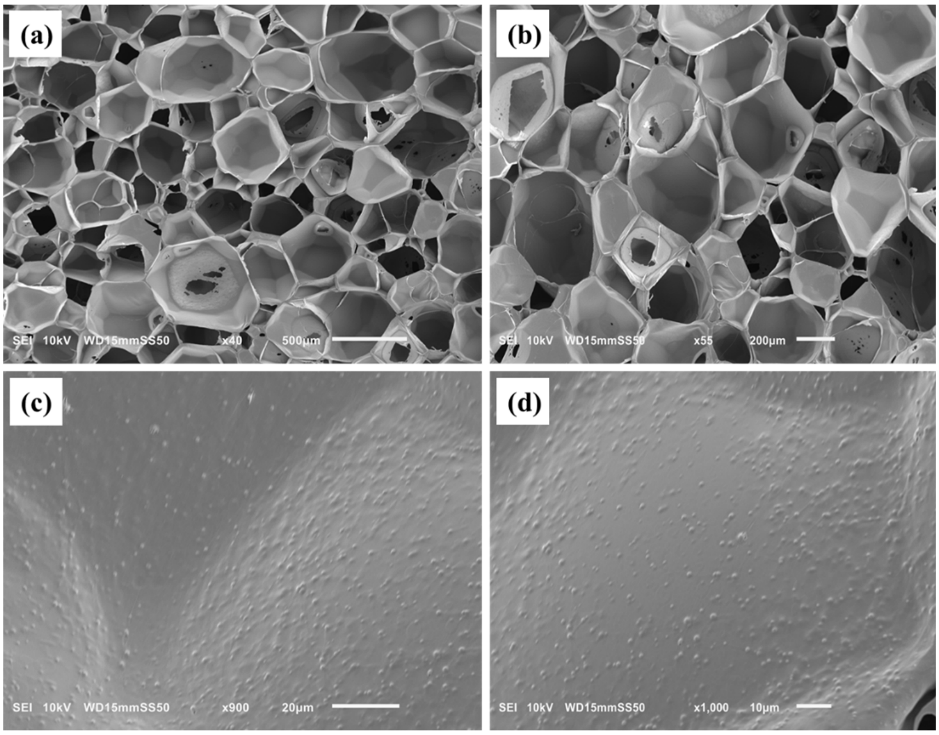

Figure 1S. SEM images of the foams of PLA/PA nanoblends (a and b) with the PA nanodroplets reinforced cell walls of PLA (c and d).

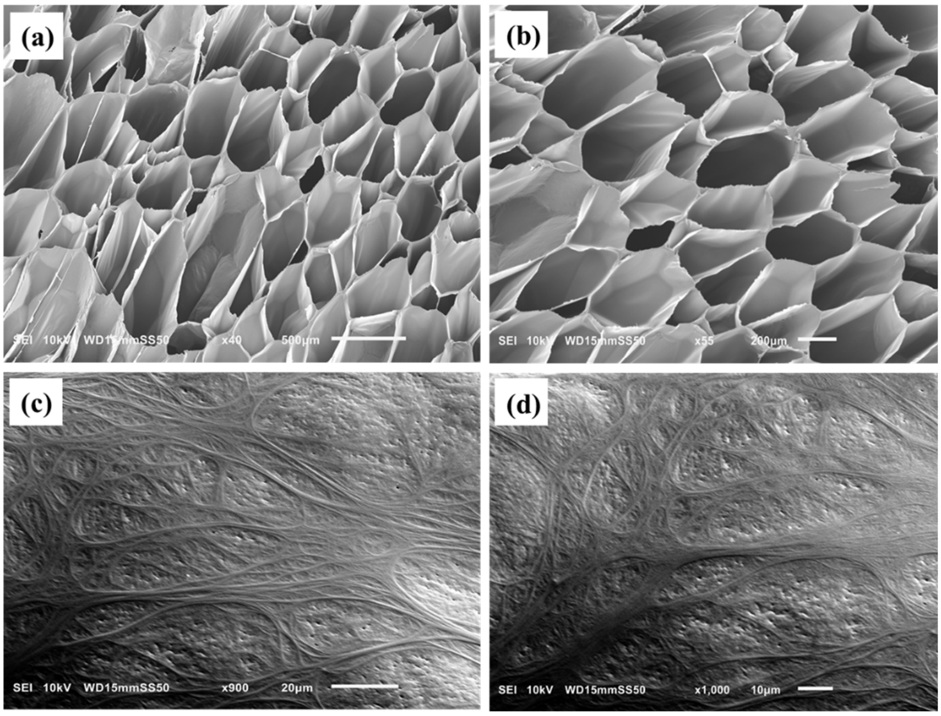

Figure 2S. SEM images of the foams of PLA/PA in-situ generated composites (a and b) with the PA nanofibers reinforced cell walls of PLA (c and d).

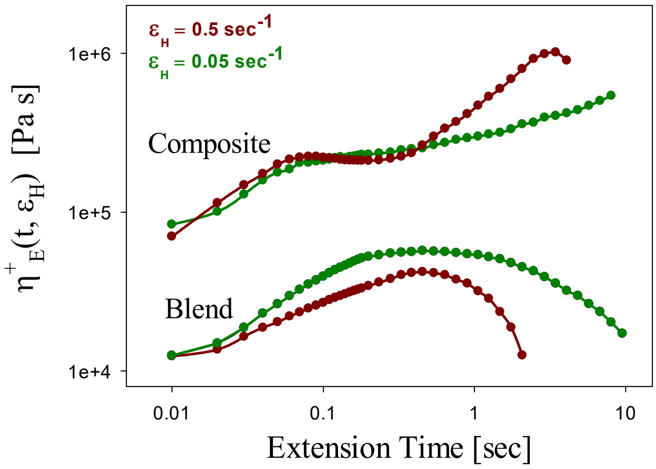

Figure 3S. Time-dependence of the tensile stress growth coefficient, $\eta^{+}_{E}(t, \varepsilon_H)$, for PLA/PA nanoblend and in situ generated composite.

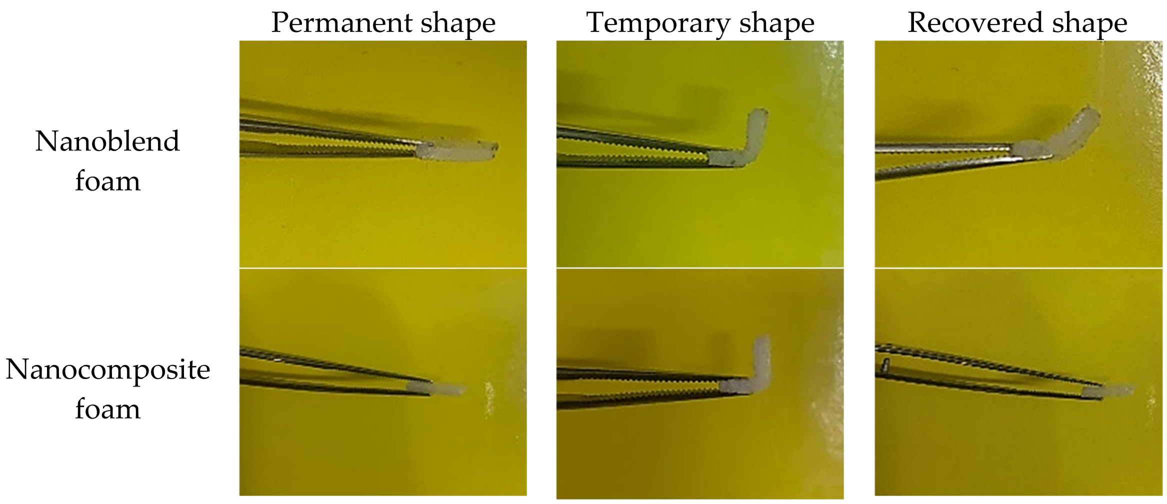

Figure 4S. Illustration of the shape memory behaviour of PLA/PA nanoblend and in-situ generated composites

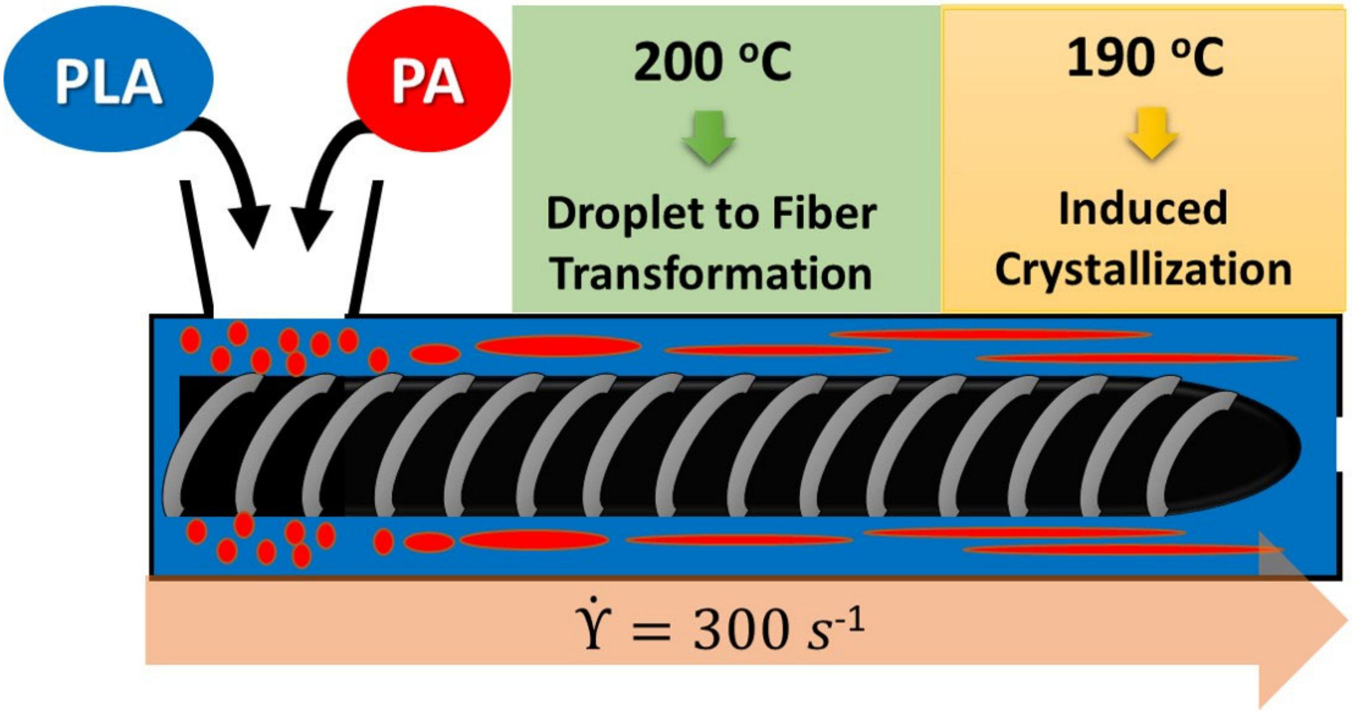

Figure 5S. Schematic illustrating the transformation of droplets into fibers and their stabilization under high shear rate during extrusion.

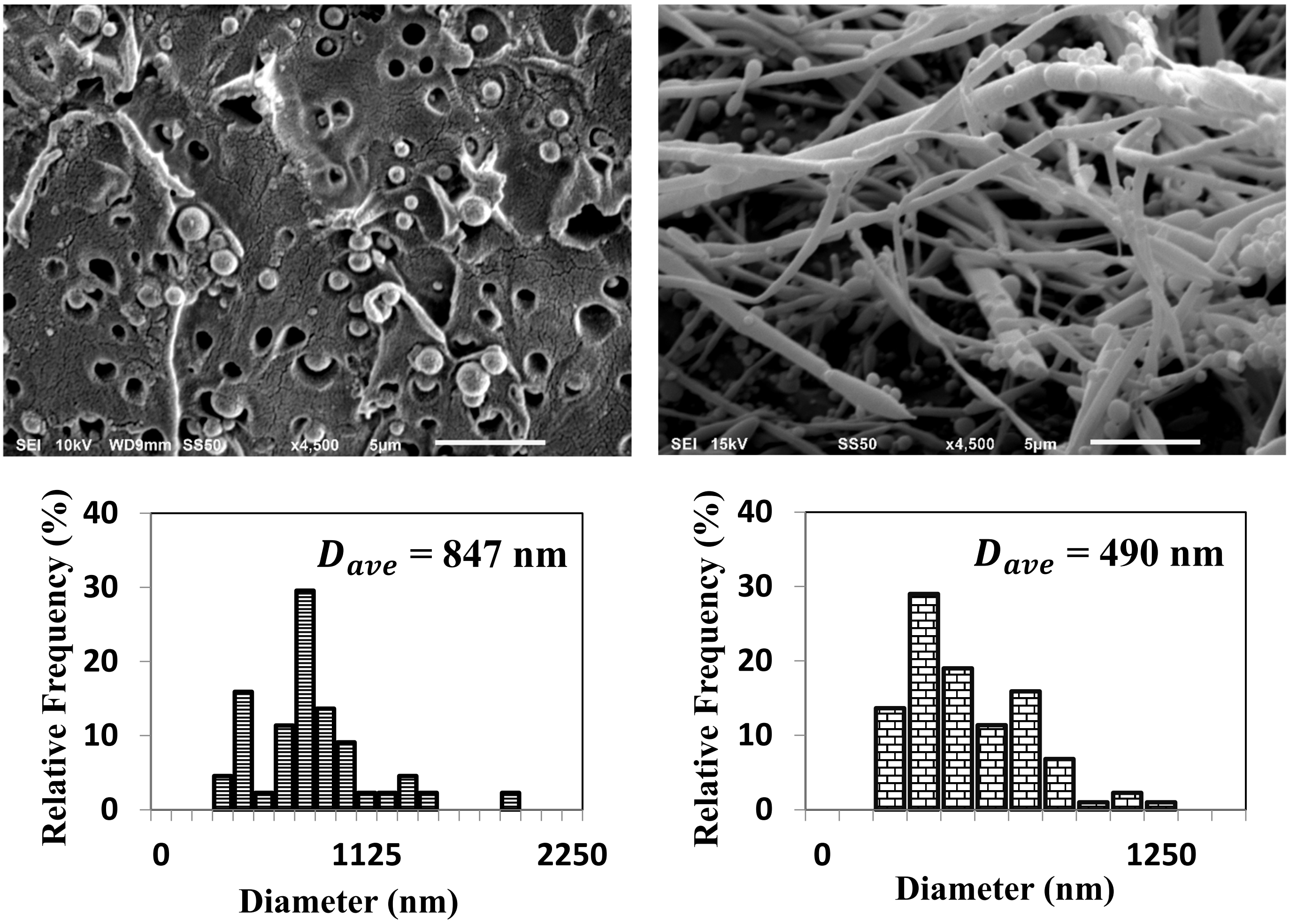

Figure 6S. SEM images of PLA/PA nanoblend (left) and in-situ generated composites (right) at higher magnification of 4500X and histograms of PA particle size distribution.
